# Perinatal outcomes in women with lower-range elevated blood pressure and stage 1 hypertension: insights from the Kaya health and demographic surveillance system, Burkina Faso

**DOI:** 10.1186/s12889-023-17424-7

**Published:** 2023-12-19

**Authors:** Franck Garanet, Sékou Samadoulougou, Calypse Ngwasiri, Abou Coulibaly, Fatou B.Sissoko, Vincent N. Bagnoa, Adama Baguiya, Seni Kouanda, Fati Kirakoya-Samadoulougou

**Affiliations:** 1 Centre National de la Recherche Scientifique et Technologique (CNRST), Institut de Recherche en Sciences de la Santé (IRSS), Département Biomédical et Santé Publique, Ouagadougou, Burkina Faso; 2 Centre de Recherche en Epidémiologie, Biostatistiques et Recherche Clinique, Ecole de Santé Publique, Université Libre de Bruxelles, Brussels, Belgique; 3 Laboratoire de Santé Publique (LASAP), Université Ouaga1 Joseph Ki-Zerbo, Ecole Doctorale Science de la Santé (ED2S), Ouagadougou, Burkina Faso; 4 Centre for Research On Planning and Development (CRAD), Laval University, Quebec, G1V 0A6 Canada; 5 Evaluation Platform On Obesity Prevention, Quebec Heart and Lung Institute, Quebec, G1V 4G5 Canada; 6 Institut Africain de Santé Publique (IASP), Ouagadougou, Burkina Faso

**Keywords:** 2017 ACC, AHA criteria, Elevated blood pressure, Stage 1 hypertension, Perinatal outcomes, Kaya

## Abstract

**Background:**

The impact of lower thresholds for elevated blood pressure (BP) on adverse perinatal outcomes has been poorly explored in sub-Saharan African populations. We aimed to explore the association between lower BP cutoffs (according to the 2017 American College of Cardiology/American Heart Association [ACC/AHA] criteria) and adverse perinatal outcomes in Kaya, Burkina Faso.

**Methods:**

This retrospective cohort study included 2,232 women with singleton pregnancies between February and September 2021. BP was categorized according to the ACC/AHA criteria and applied throughout pregnancy. A multivariable Poisson regression model based on Generalized Estimating Equation with robust standard errors was used to evaluate the association between elevated BP, stage 1 hypertension, and adverse perinatal outcomes, controlling for maternal sociodemographic characteristics, parity, and the number of antenatal consultations, and the results were presented as adjusted risk ratios (aRRs) with corresponding 95% confidence intervals (CIs).

**Results:**

Of the 2,232 women, 1000 (44.8%) were normotensive, 334 (14.9%) had elevated BP, 759 (34.0%) had stage 1 hypertension, and 139 (6.2%) had stage 2 hypertension. There was no significant association between elevated BP and adverse pregnancy outcomes. Compared to normotensive women, women with elevated BP had a 2.05-fold increased risk of delivery via caesarean section (aRR;2.05, 95%CI; 1.08–3.92), while those with stage 1 hypertension had a 1.41-fold increased risk of having low birth weight babies (aRR; 1.41, 95%CI; 1.06–1.86), and a 1.32-fold increased risk of having any maternal or neonatal adverse outcome (aRR; 1.32, 95%CI; 1.02–1.69).

**Conclusions:**

Our results suggest that the risk of adverse pregnancy outcomes is not increased with elevated BP. Proactive identification of pregnant women with stage 1 hypertension in Burkina Faso can improve hypertension management through enhanced clinical surveillance.

**Supplementary Information:**

The online version contains supplementary material available at 10.1186/s12889-023-17424-7.

## Background

Traditionally, hypertension in pregnancy has been defined as a systolic blood pressure (SBP) of at least 140 mmHg or a diastolic blood pressure (DBP) of at least 90 mmHg, or both [1]. Hypertension, as defined by this threshold, increases the risk of adverse maternal and fetal outcomes such as preeclampsia, placental abruption, preterm delivery, fetal growth restriction, and stillbirth [2, 3].

In 2017, the American College of Cardiology (ACC) and American Heart Association (AHA) redefined hypertension with lower diagnostic BP thresholds due to accumulating evidence showing increased cardiovascular morbidity and mortality among adults with lower BP levels. Therefore, more stringent average BP cut-offs have been proposed to define elevated BP as SBP 120–129 mmHg and DBP < 80 mmHg; stage 1 hypertension as SBP 130–139 mmHg or DBP 80–89 mmHg; and stage 2 hypertension as SBP ≥ 140 mm Hg or DBP ≥ 90 mmHg, or both [4]. Although obstetric guidelines have yet to adopt these new definitions of hypertension in pregnancy, several studies have shown that elevated BP and stage 1 hypertension are associated with an increased risk of adverse obstetric perinatal outcomes [5–11], and therefore, support the new ACC/AHA guidelines. Some of these studies demonstrated an improvement in the identification of women at high risk of developing gestational diabetes, preeclampsia, and preterm birth using lower BP cut-offs [6, 7]. However, nearly all the evidence supporting the use of these newer thresholds comes from high-income settings and is based on retrospective, routinely collected data. The few studies conducted in low- income and middle-income countries (LMICs) that have assessed the impact of using the new ACC/AHA BP guidelines on perinatal outcomes have shown varying results.

A prospective analysis of more than 20,000 pregnant women from three LMICs (India, Mozambique, and Pakistan) revealed that neither elevated BP nor stage 1 hypertension was associated with maternal, fetal, or neonatal mortality or morbidity or adverse composite outcomes. The authors, therefore, supported retaining the current diagnostic thresholds for hypertension in pregnancy (≥ 140/90 mm Hg) [12]. However, another prospective study in South Africa that assessed BP on entry into antenatal care reported that among 1,116 women, an additional 37.1% were classified as having abnormal BP according to the ACC/AHA criteria. In addition, pregnant teenagers in the same setting were more likely to have eclampsia at BP values below 140/90 mmHg [13, 14]. Across other countries in sub-Saharan Africa (SSA), the levels of exposure to hypertensive disorders and perinatal outcomes by integrating lower thresholds for hypertension have not been sufficiently explored, and the implications for risk stratification, ultrasound assessment of the fetus, and treatment of elevated BP are unknown.

Taken together, these controversial findings and lack of data emphasize the importance of having more contextual data on the impact of lower BP cut-offs on adverse perinatal outcomes in SSA and highlight the need to investigate the effects of elevated BP and stage I hypertension on perinatal outcomes. To fill this gap, we compared maternal and neonatal outcomes in women with different blood pressure levels (elevated BP, stage I hypertension, and stage 2 hypertension) to those in normotensive women in a large, diverse cohort of pregnant women from the Kaya Health and Demographic Surveillance System (HDSS) in Burkina Faso.

## Methods

### Study setting and population

This retrospective analysis assessed data routinely collected by the Kaya HDSS. The Kaya HDSS serves as a platform for assessing health interventions and chronic disease indicators in a health district, aiming to monitor changes over time, evaluate health programs, and provide a basis for policy decisions and capacity building. Situated in the north-central region of Burkina Faso, the Kaya HDSS catchment area encompasses 7 urban sectors in Kaya and 18 villages in the rural vicinity. The town of Kaya has both private and public health centers and 1 regional hospital (RH). In 2021, the maternal mortality rate per 100,000 was 5.2 in the Kaya district, 395 in Kaya RH, and 154.6 on a national scale. Additionally, the number of neonatal deaths recorded that year was 21 in the Kaya district, 19 in the Kaya RH, and 5,868 nationwide [15].

**Fig. 1:**
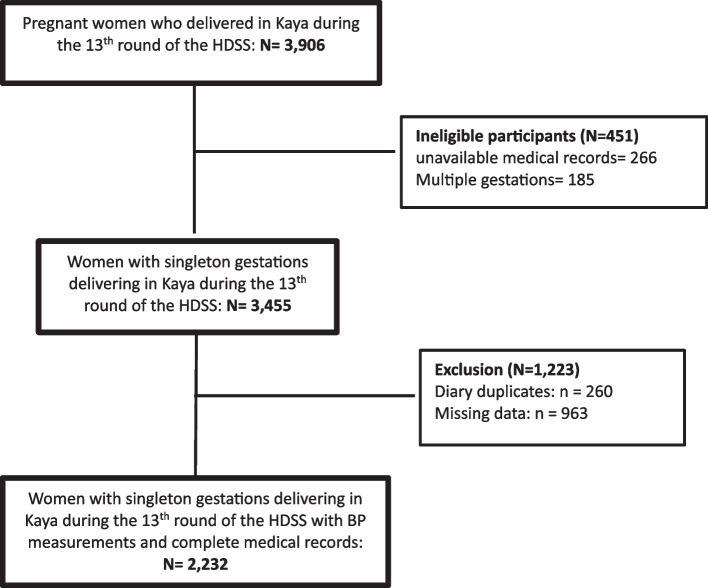
Population flowchart of pregnant women in Kaya during the 13th round of the Health and Demographic Surveillance System (HDSS), 2021

The study population consisted of all women of childbearing age who were residing in the Kaya HDSS catchment area during the 13th round of collection which took place from February to September 2021. Women who had given birth at the Kaya HDSS site since the last data collection phase and had undergone at least two antenatal consultations with an available antenatal consultation log at the time of data collection were included in this study. Women with twin pregnancies and those with incomplete or unusable health records were excluded (Fig. 1). Ethical approval was obtained from the Health Research Ethics Board of Burkina Faso (2021–07-165).

### Data collection and measures

Data were extracted from the consultation logs for pregnant women. The data collection procedure was part of the ongoing effort to monitor the resident population for vital events, including pregnancy outcomes and related maternal and infant morbidity and mortality. Details of the collection procedure and methods have been previously described [16]. Briefly, during the data collection process, households were visited by data collectors equipped with tablets that contained information about households visited during the previous data collection phase. All vital events that had occurred since the last visit were recorded. For each vital event, a specific questionnaire was administered to the appropriate household respondent by the data collectors. At the end of the data collection, the data were transferred from the tablets to a centralized server.

Baseline demographic and clinical data included maternal age, wealth index, place of residence, level of education, parity, number of antenatal consultations, and information about home deliveries. Place of residence and level of education were included to account for related social and cultural constructs that impact individual life experiences and health [16]. BP measurements throughout pregnancy were routinely performed by the medical staff using upper arm cuffs of appropriate size for each patient. The primary exposure was the ACC/AHA BP category, assigned as the highest SBP values and/or the highest DBP values from at least 2 separate dates. Elevated BP was defined as having SBP of 120–129 mmHg on at least two different dates, while stage 1 hypertension was defined as SBPs of 130–139 mmHg at least two distinct dates or DBPs of 80–89 mmHg at least two distinct dates. Patients with a prior diagnosis of chronic hypertension were categorized as having stage 2 hypertension regardless of their BP, and the remaining women were considered normotensive.

### Outcome

The primary outcome assessed was a composite of maternal, fetal, and neonatal outcomes, including cesarean deliveries, perinatal mortality, preterm birth, low birth weight, neonatal intensive care unit (ICU) admission, and poor Apgar scores at birth and miscarriages. Adverse perinatal outcome was defined as the presence of at least one adverse event [12]. Maternal, fetal, and neonatal outcomes were analyzed separately (Table 1).

**Table 1:** Definitions of the primary outcomes

Variable	Definition	Variable categorization
Low birth weight	Birth weight < 2500 g	Yes = 1 and No = 0
Poor Apgar score	Apgar score < 7	Yes = 1 and No = 0
Perinatal mortality	Stillbirth (infant death in utero at or during the intrapartum period [≥ 28 weeks of gestation]) and early neonatal death (within the first week of birth)	Yes = 1 and No = 0
Preterm birth	Birth before 37 completed weeks of gestation but after 28 weeks of gestation	Yes = 1 and No = 0
Neonatal ICU admission	Admission of the infant for reanimation	Yes = 1 and No = 0
Cesarean delivery	Delivery of a baby via a surgical incision on the mother's abdomen and uterus	Yes = 1 and No = 0
Miscarriage/abortion	Pregnancy loss at < 20 weeks of gestation	Yes = 1 and No = 0
Adverse perinatal outcome (composite variable)	Presence of at least one adverse event	Yes = 1 and No = 0

*ICU* intensive care unit

A priori selection of covariates was done for factors associated with an increased risk of adverse perinatal outcome and included maternal age, wealth index (poor, less poor, middle, less rich, or rich), place of residence (rural or urban), level of education (less than primary and at least primary education), parity (primiparity or multiparity), number of antenatal consultations (1–3 or 4–8), and home deliveries (yes or no). Maternal age was categorized as < 20, 20–35 and > 35 years because advanced maternal age is associated with an increased risk of hypertensive disorders during pregnancy [17].

### Statistical analysis

The distribution of the participants’ sociodemographic and clinical characteristics was summarised, and a descriptive analysis was presented. The association between BP groups and maternal characteristics was assessed using Pearson's chi-square test for categorical variables.

To evaluate the association between elevated BP, stage 1 hypertension, and stage 2 hypertension in comparison to the normotensive group (used as the reference) for adverse composite maternal, fetal, and neonatal outcomes, as well as individual outcomes such as Cesarean deliveries, perinatal mortality, preterm birth, low birth weight, neonatal ICU admissions, and poor Apgar scores at birth, and miscarriages, we employed a Poisson regression model based on Generalized Estimating Equation with robust standard errors.

The results were presented as unadjusted and adjusted risk ratios (RRs) with corresponding 95% confidence intervals (Cis). The statistical threshold for significance was *P* < 0.05, and all analyses were performed using STATA version 17.0.

Reporting adhered to the Strengthening Research for Observational Studies guidelines.

## Results

Of the 3,455 women with singleton pregnancies who gave birth in Kaya during the 13th round of HDSS data collection, approximately 2 out of every 3 women met the inclusion criteria, resulting in a final analysis cohort of 2,232 women (Fig. 1). A comparative analysis of characteristics between participants and non-participants revealed significant differences. Notably, most non-participants were classified as very poor, had undergone between 1 to 3 ANC consults, and had home deliveries (supplementary Table 2).

Overall, the participant women were typically enrolled in ANC either in the late first or early second trimester of pregnancy. About half of these women maintained normal BP throughout their pregnancies. Most of the women were aged 35 years or younger, resided in urban areas, had normal pre-pregnancy weights, attended at least 3 ANC visits, and delivered in a hospital setting (Table 2).

**Table 2:** Baseline characteristics of study participants according to ACC/AHA BP categories (*N* = 2,232)

	**Normotension n (%) 1,000 (44.8)**	**Elevated BP n (%) 334 (14.9)**	**Stage1 Htn n (%) 759 (34.0)**	**Stage 2 Htn n (%) 139 (6.2)**
**Maternal age (mean ± sd)**	28.6 ± 6.8	26.9 ± 7.1	27.9 ± 7.6	29.3 ± 8.2
Maternal age				
13- 19 years	71 (7.1)	41(12.3)	82 (10.8)	13 (9.4)
20–35 years	745 (74.5)	246 (73.7)	553 (72.9)	93 (66.9)
> 35 years	184 (18.4)	47 (14.1)	124 (16.3)	33 (23.7)
**Wealth index**				
Very poor	139 (13.9)	49 (14.7)	119 (15.7)	9 (6.5)
Poor	210 (21.0)	69 (20.7)	130 (17.1)	31 (22.3)
Middle	238 (23.8)	80 (23.9)	170 (22.4)	33 (23.7)
Rich	193 (19.3)	70 (21.0)	165 (21.7)	29 (20.9)
Very rich	220 (22.0)	66 (19.8)	175 (23.1)	37 (26.6)
**Place or residence**				
Urban	538 (53.8)	194 (58.1)	448 (59.1)	98 (70.5)
Rural	462 (46.2)	140 (41.9)	311 (40.1)	41 (29.5)
**Level of education**				
None	708 (70.1)	220 (65.8)	504 (66.4)	87 (62.4)
At least primary	293 (29.9)	114 (34.2)	255 (33.6)	52 (37.6)
**Parity**				
Nulliparous	765 (76.5)	212 (63.5)	493 (64.9)	99 (71.2)
Multiparous	235 (23.5)	122 (36.5)	266 (35.1)	40 (28.8)
**Antenatal consultations**				
[1-3]	378 (37.8)	127 (38.0)	203 (26.7)	27 (19.4)
[4-8]	622 (62.2)	207 (62.0)	556 (73.3)	112 (80.6)
**Home deliveries**				
Yes	5 (0.5)	0 (0.0)	3 (0.4)	0 (0.0)
No	995 (99.5)	334 (100.0)	759 (99.6)	139 (100.0)

If the stage 1 hypertension category cutoffs were used as the new threshold for diagnosing hypertension in pregnancy, an additional 759 (34%) out of the 2,232 women would be diagnosed. Similarly, if the elevated BP cutoffs were applied, an additional 334 (15%) women would be diagnosed

*ACC/AHA* American College of Cardiology/American Heart Association, *BP* blood pressure, *Htn* hypertension

Tables 3 and 4 present the primary and secondary outcomes, stratified by the ACC/AHA BP categories.

**Table 3:** Adverse maternal and fetal/neonatal outcomes with ACC/AHA BP classifications (*N* = 2,232)

Variables	**Normotension (*n* = 1,000)**	**Elevated BP (*n* = 334)**	**Stage1 Htn (*n* = 759)**	**Stage 2 Htn (*n* = 139)**	***P*-Value**
	**Frequency (%)**	**Frequency (%)**	**Frequency (%)**	**Frequency (%)**	
**Adverse perinatal outcome (composite outcome)**	101 (10.1)	31 (9.3)	109 (14.4)	20 (14.4)	**0.014**
**Cesarean section**	20 (2.0)	14 (4.2)	12 (1.6)	10 (7.2)	** < 0.001**
**Low birth weight**	74 (7.4)	20 (6.0)	78 (10.3)	16 (11.5)	**0.029**
**Neonate ICU admission**	26 (2.6)	8 (2.4)	27 (3.6)	4 (2.9)	0.62
**Prematurity**	2 (0.2)	3 (0.9)	4 (0.5)	0 (0.0)	0.27
**Poor Apgar score**	5 (0.5)	5 (1.5)	7 (0.9)	1 (0.7)	0.35

*ACC/AHA* American College of Cardiology/American Heart Association, *BP* blood pressure, *Htn* hypertension

**Table 4:** Adverse perinatal outcomes with elevated blood pressure, stage 1, and stage 2 hypertension compared with normotension in Kaya (*N* = 2,232)

Outcome	Normotension (*n* = 1000)	Elevated BP (*n* = 334)	Stage 1 Htn (*n* = 759)	Stage 2 Htn (*n* = 139)	*P*-value^*^
**Maternal Composite**					**0.038**
RR (95% CI)	1	0.88 (0.64–1.21)	1.28 (1.03–1.59)	1.69 (1.28–2.23)	
aRR (95% CI)	1	0.86 (0.60–1.25)	1.32 (1.02–1.69)	1.79 (1.89–2.48)	
**Caesarean section**					** < 0.001**
RR (95% CI)	1	1.99 (1.04–3.81)	0.72 (0.36–1.45)	3.62 (1.78–7.31)	
aRR (95% CI)	1	2.05 (1.08–3.92)	0.72 (0.35–1.44)	3.08 (1.52–6.25)	
**Low birth weight**					**0.036**
RR (95% CI)	1	0.86 (0.55–1.35)	1.37 (1.02–1.84)	1.48 (1.04–2.45)	
aRR (95% CI)	1	0.74 (0.48–1.16)	1.41 (1.06–1.86)	1.53 (1.01–2.75)	
**Neonate ICU admission**					0.74
RR (95% CI)	1	0.90 (0.41–1.97)	1.42 (0.84–2.41)	1.37 (0.54–3.53)	
aRR (95% CI)	1	0.82 (0.37–1.81)	1.23 (0.72–2.09)	1.16 (0.45–2.99)	
**Prematurity**					0.33
RR (95% CI)	1	2.93 (0.59- 14.44)	2.65 (0.66–10.57)		
aRR (95% CI)	1	2.75 (0.54–13.91)	2.75 (0.67–11.27)		
**Poor Apgar score**					0.14
RR (95% CI)	1	3.54 (1.08–11.55)	1.84 (0.58–5.78)	1.42 (0.17–12.04)	
aRR (95% CI)	1	4.49 (1.23–16.41)	1.86 (0.53–6.49)	2.11 (0.22–19.79)	

aRRs and *p-*value are adjusted for age, place of residence, parity, and number of antenatal consultations

*BP* blood pressure, *Htn* hypertension, *RR* risk ratio, *aRR* adjusted risk ratio, *CI* confidence interval

In general, when compared to women with normal blood pressure, those with stage 1 and stage 2 hypertension had significantly higher proportions of adverse perinatal outcomes (Table 3). Specifically, in the elevated BP group, the proportions of Cesarean section and poor Apgar scores were higher compared to the normotensive group. However, women with stage 1 and stage 2 hypertension did not exhibit a statistically significant increase in the occurrence of these events when compared to normotensive women. Furthermore, the proportion of low birth weight was higher among women with stage 1 and stage 2 hypertension, whereas it was lower in women with elevated BP in comparison to women with normal BP.

The likelihood of experiencing any maternal or neonatal adverse outcome was significantly higher among women with stage 1 hypertension (adjusted risk ratio [aRR]; 1.32, 95%CI; 1.02–1.69) and stage 2 hypertension (aRR; 1.60, 95%CI; 1.10–2.33) when compared with normotensive women. However, this increased risk was not observed among women with elevated BP.

Compared with normotensive women, those with elevated BP had a 2.05-fold higher risk of delivering via Cesarean section (aRR;2.05, 95%CI; 1.08–3.92), while women with stage 2 hypertension had a 3.08-fold higher risk Cesarean delivery (aRR; 3.08, 95% CI; 1.52–6.25). Women with stage 1 hypertension, on the other hand, did not exhibit a significantly higher risk of Cesarean deliveries when compared to those with normal BP.

Women with stage 1 hypertension had a 1.41-fold higher risk of giving birth to babies with low birth weight compared to normotensive women (aRR; 1.41, 95%CI; 1.06–1.86) whereas women with stage 2 hypertension had a 1.53-fold higher risk of having babies with low birth weight compared to those with normal BP (aRR; 1.53, 95% CI; 1.01–2.75).

The risks of neonatal ICU admission, prematurity, and poor APGAR scores at birth did not significantly differ among the different BP groups after multivariable analysis (Table 4).

## Discussion

To our knowledge, this is the first study in Burkina Faso to report on the impact of revised BP thresholds on perinatal outcomes. In this retrospective cohort study, we observed that adverse perinatal outcomes were more common among pregnancy subgroups with BP thresholds previously categorized as normal. The proportion of adverse events was significantly higher in women with stage 1 and stage 2 hypertension compared to normotensive women. However, no significant difference in adverse outcome was observed in women with elevated BP when compared to normotensive women.

The prevalence of hypertension in our study was 6.2% based on the current guidelines. However, if the new ACC/AHA criteria were applied, 15% more women would be categorized as having abnormal blood pressure if elevated BP cutoffs were used, and 34% more women would be diagnosed with hypertension if stage 1 hypertension cut-offs were used, in addition to those already identified as hypertensive according to the current guidelines (i.e., stage 2 hypertension in this study). This increase in the proportion of women diagnosed with hypertension depending on the chosen cutoff would significantly impact the healthcare system, necessitating increased resources for secondary prevention, including skills, diagnostic tests, CV risk stratification and the potential need for low-dose aspirin.

Compared to normotensive women, those with elevated BP had a 2.05-fold increased risk of delivering via caesarean section and a 4.49-fold higher risk of poor Apgar scores at birth. Women with stage 1 and stage 2 hypertension had a 1.41-fold increased risk and a 1.53-fold higher risk of giving birth to low-birth weight babies, respectively. Our findings revealed more adverse outcomes (cesarean section and poor apgar scores) in women with elevated BP, and for both outcomes, women with stage 1 hypertension did not have a higher risk when compared to normotensive women. This suggests an absent dose–response relationship between higher blood pressure categories and increased risk for adverse perinatal outcomes. Furthermore, the proportion of any adverse maternal/neonatal outcome (composite outcome) was lower in women with elevated BP compared to normotensive women. Taken together, these suggest that an association between elevated BP and adverse outcomes is not very likely based on the study data.

Our findings mirror those of the secondary analysis of Community-Level Interventions for Pre-eclampsia (CLIP) trial data from other low–resource settings, where neither elevated BP nor stage 1 hypertension increased the risk of adverse maternal, fetal, or neonatal outcomes when compared with normotensive women [12].

The study results contrast those of numerous studies that reported an increased risk of adverse pregnancy outcomes in women with antenatal elevated BP or stage 1 hypertension [5, 8, 11, 18–21]. Possible explanations for this difference include our analysis of unselected pregnant women compared to previous studies focusing on nulliparous women [7, 22, 23], fewer baseline characteristics used for adjusting risk ratios (RRs), and differences in outcome assessment. Furthermore, our analysis included BP measurements taken throughout pregnancy, mainly after 20 weeks of gestation, in contrast to studies that restricted BP observations to women at less than 20 weeks of gestation [5, 7, 11, 22].

The absence of an increased risk of neonatal ICU admission, poor Apgar scores, and premature delivery in women with abnormal BP was not explained by differences in demographic or clinical covariates in our study. Additionally, we observed no perinatal death, abortion, or miscarriage possibly due to the fact that consultation logs were destroyed in the event of maternal or infant death.

Although the association between elevated BP and adverse outcomes is less likely in this study, our findings highlight that the higher risks associated with the new ACC/AHA BP categories stage 1 hypertension should not be neglected by prenatal care providers in LMICs and, as noted by previous authors, may extend into the postpartum period [11]. Interventions that increase the awareness of raised BP, promote healthy weight gain and lifestyle, and provide closer BP monitoring have been shown to be effective during pregnancy [7, 23, 24].

Our findings should be considered in the context of the study’s limitations and strengths. The retrospective cohort study design with a relatively small sample size was limited by the available data in women’s antenatal records and databases, resulting in a smaller group with sufficient BP data for use in the assessment of the ACC/AHA criteria. Additionally, as BP was measured during routine clinic visits, these measures may not have been as rigorous as they would have otherwise been in a prospective study. Other factors, such as medication use (e.g., aspirin and antihypertensives), preeclampsia, placental abruption, gestational diabetes, intrauterine growth restriction, hospitalization, and postpartum readmission were not easily extractable, and the impact of lower BP thresholds on early pregnancy loss could not be ascertained. The exclusion of women with missing data raises concerns about potential selection bias, as the excluded women may be at higher risk of both blood pressure issues and adverse perinatal outcomes. Data collection from the consultation logs of women who have given birth provides no data on abortions or miscarriages. Finally, as this was a study from a single health district, the generalisability to other institutions must be carefully considered. We attempted to minimize these limitations by using multiple BP measurements throughout pregnancy and robust statistical methods to adjust for confounding factors.

On the positive side, our study included a diverse sample of singleton pregnancies and births in Burkina Faso and is the first of its kind in the country to explore the implementation of the 2017 ACC/AHA definition of hypertension, and its impact on maternal and fetal outcomes. Additionally, this study included all pregnant women with available data, ensuring a comprehensive representation of the population of pregnant women at the Kaya HDSS site. To comprehensively assess the impact of lower BP thresholds on adverse perinatal outcomes, further studies are warranted, including prospective cohorts with larger sample sizes that allow for more precise BP measurements, as well as data collection from multiple health districts on variables such as birth interval, eating habits, early pregnancy loss, medication use, pre-pregnancy weight, preeclampsia, placental abruption, gestational diabetes, intrauterine growth restriction, hospitalization, and postpartum readmission.

## Conclusions

In this cohort study, our results indicated that the association between elevated BP and adverse pregnancy outcomes was not significant, while stage 1 and Stage 2 hypertension were associated with increased risk of poor neonatal outcomes. Optimal management of hypertension in pregnant women in Burkina Faso requires a proactive approach to identify women at a heightened risk to permit healthcare professionals provide more vigilant clinical surveillance for those who may have otherwise been overlooked. Additional studies, including prospective cohorts and randomized controlled trials, are needed to validate our current outcomes and assess the potential benefits of initiating treatment in pregnant women with lower BP thresholds.

### Supplementary Information

**Additional file 1:** **Supplementary** **Table 2.** Baseline characteristics of study participants and non-participants.

## Data Availability

The datasets used and/or analysed during the current study available from the corresponding author on reasonable request.

## Abbreviations

ACC/AHA: American College of Cardiology/American Heart Association
ANC: Antenatal care
BP: Blood pressure
CI: Confidence interval
DBP: Diastolic blood pressure
HTN: Hypertension
ICU: Intensive care unit
Kaya–HDSS: Kaya health and demographic surveillance system
LMICs: Low– and middle–income countries
RH: Regional Hospital
aRR: Adjusted risk ratio
SBP: Systolic blood pressure
SSA: Sub–Saharan Africa
