# Supplementary material for: Perinatal outcomes in women with lower-range elevated blood pressure and stage 1 hypertension: insights from the Kaya health and demographic surveillance system, Burkina Faso
Source: BMC Public Health. 2023 Dec 19;23:2539. doi: 10.1186/s12889-023-17424-7 (PMC10729335; doi:10.1186/s12889-023-17424-7)
Supplement: Supplementary file 1 — Additional file 1: Supplementary Table 2. Baseline characteristics of study participants and non-participants. [file 12889_2023_17424_MOESM1_ESM.pdf]

Supplementary Table 2: Baseline characteristics of study participants and non-participants

	Inclusion (N=2,232	Exclusion (N= 1,223)	P-value*
	Frequency (%)	Frequency (%)	
**Maternal age**			0.13
13- 19	207(9.3)	95(11.1)	
20–35 years	1637 (73.3)	596 (69.9)	
>35 years	388 (17.4)	162 (19.0)	
**Wealth index**			**< 0.001**
Very rich	498 (22.3)	152 (16.1)	
Rich	457 (20.5)	196 (20.7)	
Middle	521 (23.3)	193 (20.4)	
Poor	440 (19.7)	188 (19.9)	
Very poor	316 (14.2)	217 (22.9)	
**Place of residence**			0.06
Urban	1278 (57.3)	576 (60.9)	
Rural	954 (42.7)	370 (39.1)	
**Level of education**			0.38
None	1007 (64.9)	381 (67.0)	
At least primary	544 (35.1)	188(33.0)	
**Parity**			0.98
Nulliparous	663 (29.7)	333 (29.6)	
Multiparous	1569 (70.3)	790 (70.4)	
**Antenatal consultations**			**< 0.001**
[1–3]	735 (32.9)	507 (45.2)	
[4–8]	1497(67.1)	616 (54.8)	
**Home deliveries**			**< 0.001**
Yes	8 (0.4)	42 (3.7)	

No	2224 (99.6)	1081(96.3)

* *P-value for chi-square test of proportions*
